# Investigations aimed at producing 33% efficient perovskite–silicon tandem solar cells through device simulations†

**DOI:** 10.1039/d1ra06250f

**Published:** 2021-11-19

**Authors:** Nikhil Shrivastav, Jaya Madan, Rahul Pandey, Ahmed Esmail Shalan

**Affiliations:** VLSI Centre of Excellence, Chitkara University Institute of Engineering and Technology, Chitkara University Punjab India jaya.madan@chitkara.edu.in rahul.pandey@chitkara.edu.in; BCMaterials, Basque Center for Materials, Applications and Nanostructures Martina Casiano, UPV/EHU Science Park, Barrio Sarriena s/n Leioa 48940 Spain a.shalan133@gmail.com ahmed.shalan@bcmaterials.net; Central Metallurgical Research and Development Institute (CMRDI) P. O. Box 87, Helwan Cairo 11421 Egypt

## Abstract

The conversion efficiencies for silicon-based photovoltaic devices have become stagnant, with the record conversion efficiency of 26.7% achieved in 2017. This record efficiency is also close to the theoretical Auger limit of 29.4% for single-junction silicon solar cells. Therefore, it is anticipated that further enhancement in conversion efficiency could only be achieved by adopting multijunction or tandem concepts for silicon PV devices. In this context, perovskites are widely preferred for tandem application with silicon solar cells to mitigate thermalization and non-absorbed photon losses to achieve higher conversion efficiencies. The perovskite–silicon (PVK–Si) tandem design can deliver 45.1% efficiency, and currently, this design holds a record conversion efficiency of 29.5%. Therefore, critical research and development activities are required to unlock the potential of such devices. Thus, we have designed and investigated enhanced hole extraction PVK–Si monolithic tandem solar cells with 33% power conversion efficiency (PCE) to make a humble contribution in this field. The device is facilitated with Me-4PACz and ITO-based ideal tunnel recombination junctions for current matching, with parasitic absorption losses. Detailed standalone and tandem analysis has been carried out in terms of absorber layer thickness variation, illuminated current density–voltage (*J*–*V*) curves, external quantum efficiency (EQE), energy band diagrams (EBDs), filtered spectra, filtered integrated power, current matching, and tandem PV parameters to finalize the conversion efficiency. The device constructed using a 1.68 eV perovskite top cell and 1.12 eV c-Si-based heterojunction with an intrinsic thin layer (HIT) based bottom cell showed an open-circuit voltage, *V*_OC_, of as high as 2.02 V. The comprehensive analysis of PVK–Si tandem devices reported in this work may pave the way for developing high-efficiency tandem solar cells in the future.

## Abstract 2

The conversion efficiencies for silicon-based photovoltaic devices have become stagnant, with the record conversion efficiency of 26.7% achieved in 2017.

## Graphical abstract

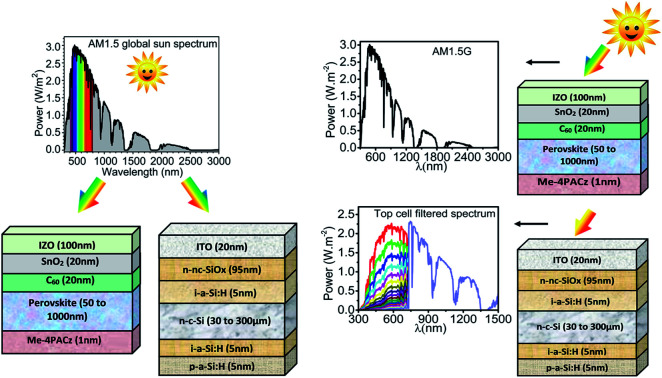

## 1. Introduction

**Fig. 1:**
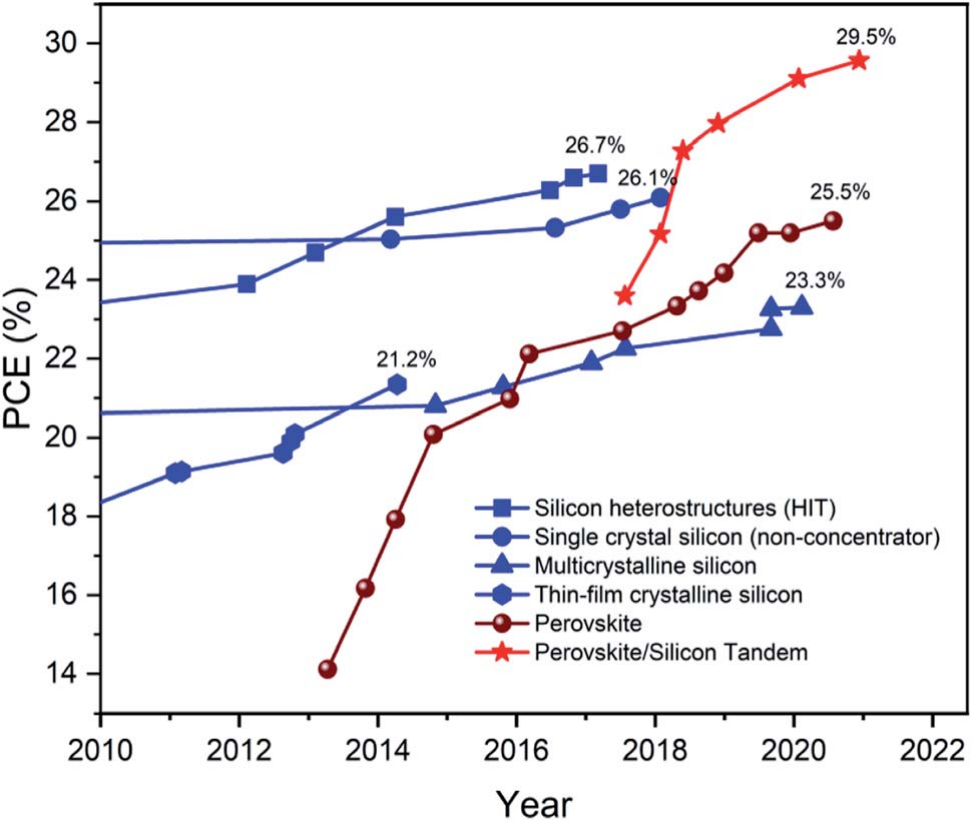
Efficiency chart for silicon, perovskite, and perovskite–silicon tandem devices in the last decade. Data derived from the efficiency table published by National Renewable Energy Laboratory (NREL).^29^

Directly converting sunlight into electricity by using semiconducting materials is termed photovoltaic (PV) technology. The adoption of PV technology relies mainly on cost, lifetime, and conversion efficiency. The fulfillment of these criteria leads to the deployment of PV technology across the globe to fulfill energy needs. However, it is never-ending demand to reduce the cost further while maintaining the performance and a lifetime.^1^ Therefore, extensive research and development have been carried out all around to achieve the objective. This resulted in a significant reduction in the levelized cost of energy under approximately 4 cents per kW per h, and this value is ten (10) times lower than what we had ten (10) years before. This improvement is not because of the enhancement in the performance but driven by development in fabrication processes and synchronic implementation at lab scale and module level. The economics of the scale also played a significant role in overall cost reduction. In the current scenario, crystalline silicon (c-Si) dominates the PV market with more than 90% market share.^2,3^ However, the performance of c-Si solar cells becomes stagnant with a record efficiency of 26.7% (ref. 4) (Fig. 1), which is near to the theoretical Auger limit of 29.4% (ref. 5) for silicon solar cells. However, further reduction in cost demands a pathway to increase the conversion efficiency, which seems to be a far-fetched objective due to the inherent losses such as thermalization and transparent *E*_g_, associated with single-junction devices.^6^ Also, considering the current market density for Si PV, one cannot deplete or replace the existing production line with some other material and technology to enhance the conversion efficiency in the near future. Therefore, to open the window for further developments, researchers coupled the silicon-based PV technology with other promising semiconducting materials to form the tandem solar cell and to improve the harvesting of electromagnetic spectrum emitting by the Sun.^7^ Tandem design concurrently mitigates the thermalization and transparent *E*_g_ loss linked to the bottom and top subcell, respectively.^8^ Further, c-Si tandem solar cells are known to have the potential to surpass the Auger limit of 29.4% for silicon solar cells as well as the SQ limit of 33% for single-junction solar cells.^6^

Initially, III–V compound semiconductors were introduced for silicon-based tandem solar cells.^9–11^ However, elevated manufacturing costs associated with III–V compound-based PV technology restricted its further development.^11^ Therefore, for the development of silicon tandem solar cells for better performance at a lower price, researchers have utilized hybrid organic–inorganic halide-based perovskite materials for the application as top subcell.^12,13^ For perovskite solar cells in a standalone application, the tremendous research effort of the researchers across the globe resulted in a recent record conversion efficiency of 25.6%,^14^ followed by previous record efficiency of 25.2% (ref. 15 and 16) (Fig. 1). The improved standalone performance also led to the development of perovskite–silicon (PVK–Si) tandem solar cells and received significant attention from researchers. This combination had performed well and had seen a massive rise in the performance in the conversion efficiency since its inception.^17–24^ It has been more than three years, but the record conversion efficiency of silicon is still the same, *i.e.*, 26.7% (Fig. 1). PVK–Si exceeded that value in 2018 and recently reflected the record conversion efficiency of 29.5% after beating the previous record efficiency of 29.15% obtained in early 2020 (Fig. 1).^24^*Helmholtz-Zentrum Berlin, German*y, *the École polytechnique fédérale de Lausanne, Switzerland*, and *Oxford Photovoltaics Limited, Oxford University Yarnton, United Kingdom*, are the main contributors to the development of PVK–Si tandem solar cells with record efficiencies.^25^ The theoretical efficiency limit for series-connected PVK–Si tandem solar cells is 45.1%,^26,27^ and researchers at Oxford PV realized 29.5% (Fig. 1) efficient PVK–Si tandem solar cells experimentally.^28^

The trend in previous research in this domain showed the possibilities to unlock the efficiency potential of PVK–Si tandem solar cells close to theoretical limits. However, it is difficult to accomplish the same by going ahead with only experimental works, since the detailed understanding of carrier dynamics, proper optimization of the absorber layer thickness and current matching, *etc.* needs to be addressed and optimized through appropriate device simulations to explore the potential of PVK–Si tandem devices.^30,31^ Therefore, to make a humble contribution in this field, we have reported the device simulations of PVK–Si tandem solar cells to achieve conversion efficiencies of 33% with 1.68 eV and 1.12 eV bandgap-based top and bottom subcells. A 29.15% efficient PVK–Si tandem solar cell reported by Ashouri *et al.*^24^ is selected to optimize the performance to unlock 33% conversion efficiencies.

The overall manuscript is divided into four subsections. The ongoing Introduction section (1) follows the Device structure and simulation methodology section (2), which thoroughly discusses simulation methods and material parameters. After that, the Result and discussion section (3) reveal the influence of the absorption layer thickness variation in both the top and bottom subcell, followed by designing PVK–Si tandem solar cells. The summary of the research work is concluded in the Conclusion section (4), along with the future scope of the work.

## 2. Device structure and simulation methodology

**Fig. 2:**
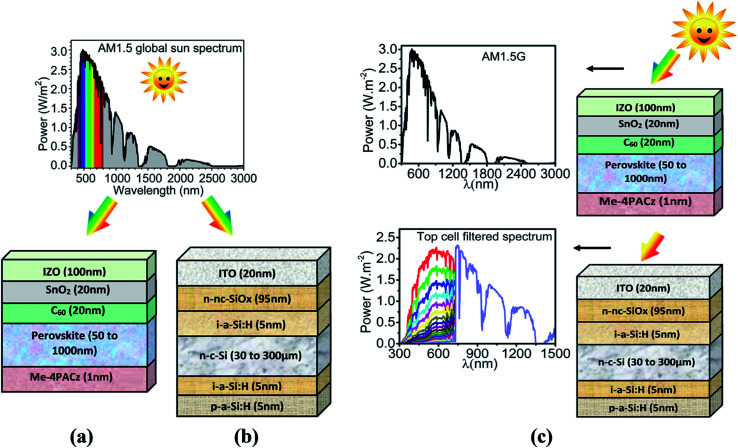
Simulated devices along with spectrums under illumination (a) schematic diagram of top subcell with AM1.5 spectrum, (b) schematic diagram of bottom subcell with AM1.5 spectrum, (c) schematic diagram of tandem cell with AM1.5 spectrum at the top subcell and filtered spectrum at bottom subcell.

The presented work used SCAPS-1D software originated in the Department of Electronics and Information System of the University of Ghent, Belgium.^32^ This software helped in simulating the device and was also used for depicting results in the form of energy band diagram (EBD), current density–voltage (*J*–*V*) curve, external quantum efficiency (EQE) curve, and the PV parameters. The simulation of the two subcells, *i.e.*, top and bottom, is performed with different materials, and their configuration is elaborated successively herewith as follows. Initially, the perovskite (*E*_g_ = 1.68 eV) material is targeted for the top cell, while c-Si (*E*_g_ = 1.12 eV) based heterojunction with intrinsic thin layer (HIT) solar cell is targeted for the bottom cell. In the previous literature, the utilized absorber layer in the top cell showed phase stability under illumination owing to the presence of Me-4PACz. The utilized HTL resulted in enhanced hole extraction, which further prevented the piling up of the carriers and nonradiative recombination at the absorber/HTL interface.^24^ The architecture for top and bottom subcells is similar to work reported by Ashouri *et al.*^24^ The top cell consists of IZO window layer, SnO_2_/C_60_ stacked electron transport layer (ETL), perovskite active layer, and self-assembled monolayer (SAM) modified with methyl group substitution ([4-(3,6-dimethyl-9*H*-carbazol-9-yl)butyl]phosphonic acid) termed as Me-4PACz as hole transport layer (HTL). Thus, the final structure of the top cell consists of IZO (100 nm)/SnO_2_ (20 nm)/C_60_ (20 nm)/perovskite (varied from 50 to 1000 nm) and Me-4PACz (1 nm). The complete structure for the perovskite top cell is shown in Fig. 2(a).

Likewise, the bottom cell consists of ITO (20 nm)/n-nc-SiO_*x*_ (95 nm)/i-a-Si:H (5 nm)/n-c-Si (varied from 30 to 300 μm)/i-a-Si:H (5 nm)/p-a-Si:H (5 nm), and the complete architecture is shown in Fig. 2(b). Standard AM 1.5 G spectrum is utilized for the standalone simulation of top and bottom subcells. The widely adopted filtered spectrum followed by the current matching strategy is used to simulate the PVK–Si tandem solar cell, as shown in Fig. 2(a–c). The results and discussion section provides a detailed discussion related to tandem simulation in terms of filtered spectrum, current matching, and construction of tandem *J*–*V* curve. An extensive literature survey is done to obtain the electrical and optical properties for all the materials used during the simulation. The obtained electrical parameters are reported in Table S1.† The interfacial defects at the heterojunctions are also considered, and information is provided in Table S2.† The neutral type bulk defects with density 1 × 10^14^ cm^−3^, 1 × 10^18^ cm^−3^, 1 × 10^14^ cm^−3^, 2 × 10^14^ cm^−3^, 1 × 10^14^ cm^−3^, 1 × 10^16^ cm^−3^, 5 × 10^11^ cm^−3^ and 5 × 10^10^ cm^−3^ are also considered for IZO, SnO_2_, C_60_, perovskite, Me-4PACz, ITO, n-nc-SiO_*x*_ and n-c-Si, respectively with single energetic distribution at the middle of the bandgap with a capture cross-section of 1 × 10^−15^ cm^2^ for both electrons and holes. In addition, three different defects such as multivalent (amphoteric defects), acceptor, and donor are also considered for intrinsic and p-type a-Si:H layers. The complete details are provided in Table S3.† SCAPS-1D numerically solves coupled Poisson's and continuity equations with dedicated boundary conditions for both holes and electrons at different interfaces and contacts to obtain the illuminated *J*–*V* curve. The fundamental equations for the optical model of SCAPS-1D and the semiconductor equations used to simulate the optoelectronic performance are in accordance with our previously published work,^33–35^ and reiteration of the same is avoided in this work.

## 3. Results and discussions

The complete result and discussion section are categorized into three different sub-sections. Initially, detailed device simulation and calibration for the perovskite (top cell) and c-Si-based HIT (bottom cell) are provided in Section 3.1. After that, both the top and bottom subcells are integrated to form the complete two-terminal monolithic tandem solar cell, whose results are provided in Section 3.2.

### 3.1. Standalone simulation and optimization of perovskite top cell

In this subsection, initially, a perovskite solar cell comprised of stacked SnO_2_/C_60_ based ETL and Me-4PACz based HTL layered device is designed, calibrated, and optimized. The architecture of the device is identical to the top cell design recently published by Ashouri *et al.*^24^ Authors developed different HTLs for top cells such as PTAA, 2PACz, and Me-4PACz and investigated the performance of the device. It has been reported that Me-4PACz based devices outperform compared to the rest of the HTL layers. The presence of Me-4PACz as HTL showed improved extraction of the light-generated carrier (hole). Also, it reflected a lower ideality factor due to better alignment of valence band edge and superior carrier mobility. Therefore, we have considered the champion device with Me-4PACz as HTL for further simulation analysis reported in this work.

**Fig. 3:**
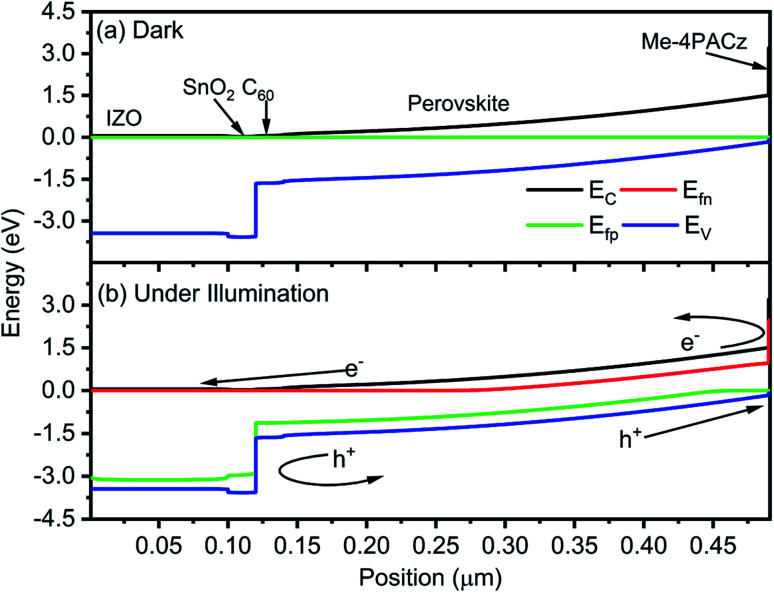
Energy band diagram of perovskite solar cell under (a) dark and (b) illumination. The data is obtained without applying external biasing.

**Fig. 4:**
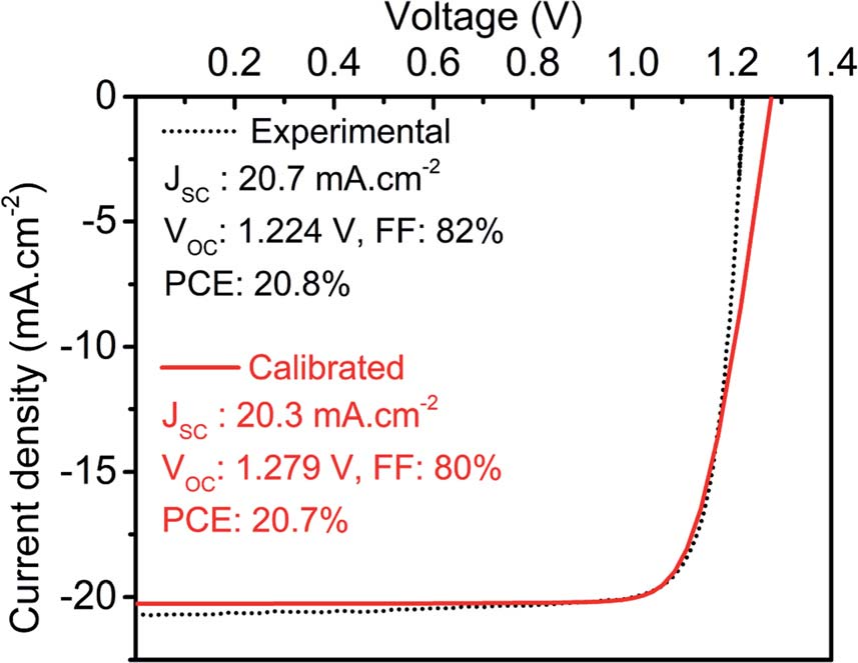
Illuminated *J*–*V* curve for calibrated (this work) and experimental top cell. The illuminated *J*–*V* data for the experimental device is derived from the literature.^24^

Further, it is hard to understand the carrier dynamics using purely experimental work. Therefore, energy band diagrams for the perovskite top subcell are obtained through device simulation and depicted in Fig. 3(a and b). To understand the generation and direction of movement for electron–hole pairs, the EBD data is obtained in both dark and illuminated conditions. A single Fermi level is observed under thermal equilibrium whereases the splitting of Fermi level is obtained under illumination, which validates the generation of electron–hole (e–h) pairs. In addition, the presence of ETL/absorber and absorber/HTL interface creates sufficient electric field to separate and collect the desired charge carrier and simultaneously prevent the movement of counterpart, as shown in Fig. 3(b). Data reported here also validates the minimum offset between the valence band of the absorber layer and HTL, which is in accordance with the experimental findings. The illuminated *J*–*V* curve and PV parameters are also obtained for the top cell and compared with experimental work, as shown in Fig. 4. The simulated results are in close proximity with the experimental work, which authenticates the calibration of the top cell design.

**Fig. 5:**
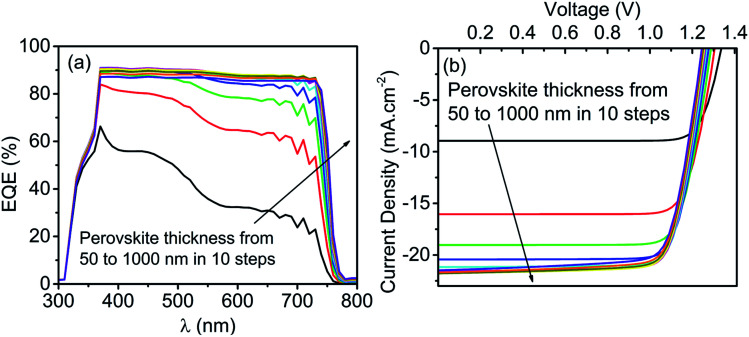
Impact of perovskite thickness (50 nm to 1000 nm) on (a) EQE and (b) *JV* curve of perovskite top cell in a standalone configuration.

After calibrating the top cell with an absorber layer thickness of 350 nm, the optimization of the device is carried out in terms of absorber layer thickness variation from 50 nm to 1000 nm in ten equal steps to enhance the conversion efficiency further. The results for thickness variation are reported in Fig. 5(a) and (b), where Fig. 5(a) shows the impact of thickness on the EQE curve, whereases the illuminated *J*–*V* curve is provided in Fig. 5(b). An increase in the thickness resulted in better photon harvesting in the absorber layer, and the same is verified through EQE reported in Fig. 5(a). The enhancement in EQE is further reflected in the *J*–*V* curve, which improves *J*_SC_ while increasing the thickness.

**Fig. 6:**
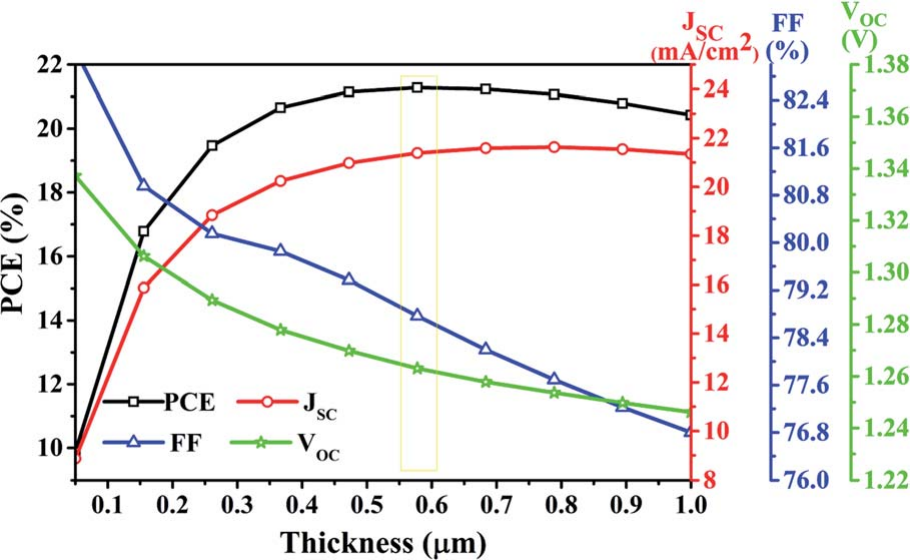
Impact of perovskite thickness (50 nm to 1000 nm) on PV parameters of top cell in a standalone configuration.

The PV parameters variation with thickness can easily be observed in Fig. 6. For an increase in thickness up to 500 nm, *J*_SC_ increases to 21.63 mA cm^−2^ owing to the enhanced absorption and consequently the generation rate. Further, an increase in thickness beyond 500 nm saturates the absorption, and hence the *J*_SC_ remains unaltered. Precisely, a marginal reduction in *J*_SC_ after 800 nm thick perovskite is obtained due to the enhanced recombination in thick perovskite. However, the inverse variation of *V*_OC_ with thickness has been obtained due to the reduced electric field strength across the thick perovskite layer. Subsequently, the reduced electric field decreases the probability of separation of generated charge carrier and reduces the *V*_OC_ (from 1.33 V to 1.24 V) and FF (from 83.24% to 76.80%) of the cell. Another factor that contributes to a reduction of FF is the increased series resistance for thick perovskite. The overall impact of *J*_SC_, *V*_OC,_ and FF have been obtained in PCE, that evidence shows that enhancement in *J*_SC_ dominates over the reduction in *V*_OC_ and FF. Consequently, PCE increases to 21.27% with the thickness of perovskite up to 577 nm and saturates after that. Precisely the PCE marginally reduces (Fig. 6) at thick perovskite (>577 nm) due to the consequences mentioned above.

### 3.2. Standalone simulation and optimization of c-Si HIT bottom cell

**Fig. 7:**
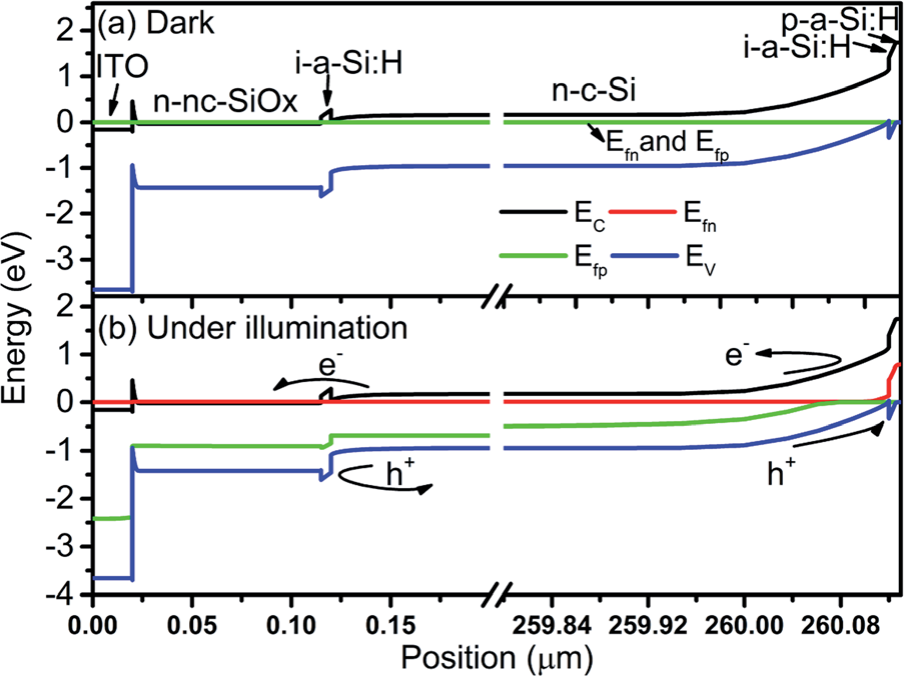
Energy band diagram of c-Si HIT solar cell under (a) dark and (b) illumination. The data is obtained without applying external biasing.

Here, the results of the bottom cell are compiled under standalone conditions. We have used a c-Si HIT solar cell in this context, as shown in Fig. 2(b), which is simulated and optimized. The initial dimension of each layer is as per the experimental work.^24^ First, to recognize the carrier dynamics, the EBD data of the solar cell is obtained and reported in Fig. 7(a and b). It is observed that the electric field near the i-a-Si:H (when adjacent to p-a-Si:H) prevents the movement of electrons. However, at the same time facilitates the collection of holes. Likewise, the electric field near the i-a-Si:H (when adjacent to n-nc-SiO_*x*_) prevents the movement of a hole and concurrently allows the transfer of electrons. After illumination, unidirectional movement and subsequent light-generated carriers validate the PV effect in the device.

**Fig. 8:**
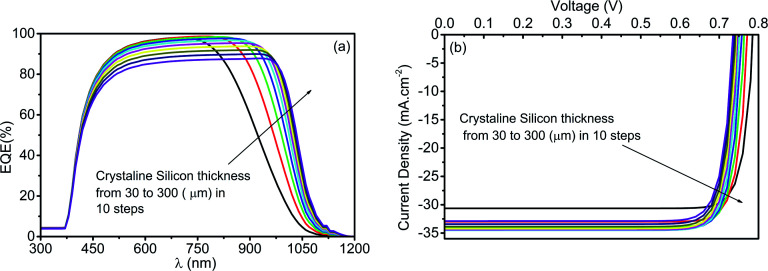
Impact of the thickness of c-Si (30 μm to 300 μm) on (a) EQE and (b) *JV* curve of the bottom cell in a standalone configuration.

Further, EQE and *JV* curves of c-Si-based HIT solar cells are obtained at the various thickness of c-Si ranging from 30 to 300 μm in 10 steps as depicted respectively in Fig. 8(a) and (b). The EQE curve shows the absorption of high-energy photons at the 30 μm thick c-Si absorber layer. While with the increase in c-Si thickness, the photons with comparatively low energy also get absorbed by the c-Si. This is evident from the penetration of the EQE curve towards the higher wavelength side. In contrast, the EQE at a low wavelength reduces with an increase in the thickness of c-Si because of the reduction in the collection probability for the thick c-Si layer.

**Fig. 9:**
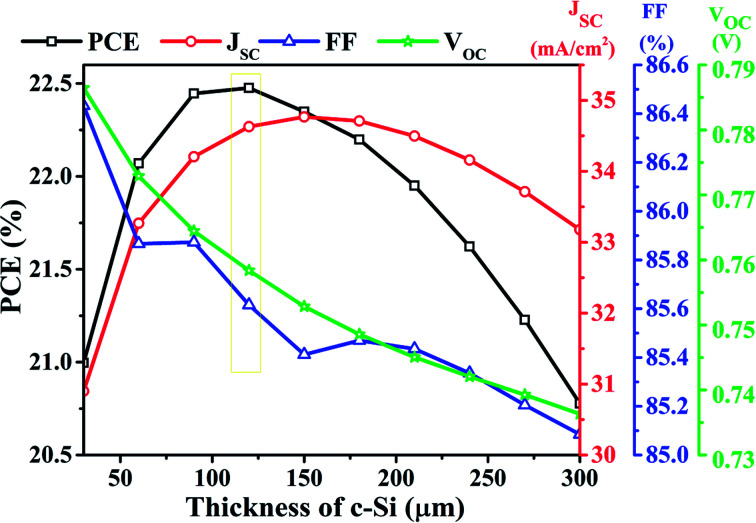
Impact of the thickness of c-Si (30 μm to 300 μm) on PV parameters of the bottom cell under standalone condition.

Fig. 9 depicts the PV parameters of the bottom cell designed with c-Si as the absorber layer at various c-Si thicknesses (30 μm to 300 μm). The plot represents the increase in *J*_SC_ from 30.89 mA cm^−2^ to 34.76 mA cm^−2^ with the rise in c-Si from 30 μm to 150 μm, due to the enhanced photon absorption followed by generation and better collection. Nevertheless, with a further increase in c-Si thickness, a downfall in *J*_SC_ has been obtained owing to the enhanced separation between the collecting interfaces, which eventually reduces the collection probability and also increases the carrier recombinations. By the same token (as that of the top cell), the FF of the bottom cell designed with c-Si reduces with an increase in c-Si thickness. Again, *V*_OC_ of the bottom cell reduces marginally with an increase in c-Si thickness because of the reduced electric field strength at thick c-Si. The cumulative impact of the abovementioned PV parameters derives the overall conversion efficiency where the highest PCE is recorded as 22.47% at 120 μm thick c-Si. Beyond 120 μm, the reduced *V*_OC_ and FF take over the charge and deteriorate the overall PCE depicted in Fig. 9.

### 3.3. Two terminal monolithic PVK–Si tandem solar cell

**Fig. 10:**
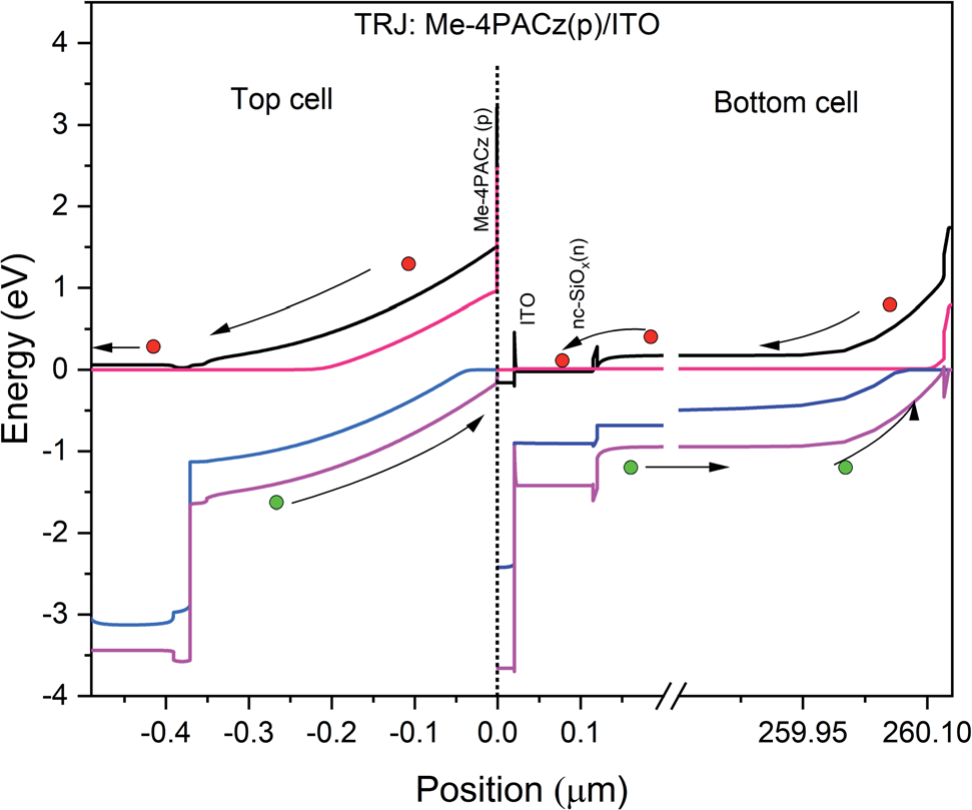
Energy band diagram of the simulated PVK–Si tandem solar cell under illumination.

After analyzing the performance of the perovskite-based top and c-Si-based bottom cell in standalone condition, this subsection reports the tandem configuration of the perovskite/c-Si as top/bottom subcell. In previous literature, two-terminal monolithic tandem solar cells are fabricated using layer by layer deposition, and top and bottom subcells are connected through interfacial tunnel recombination junction (TRJ).^17–24^ The TRJ provides the current matching between the top and bottom subcell and allows the equal current at all points, which is an essential requirement for a series-connected tandem solar cell. We understand the need for TRJ in monolithic tandem solar cells and investigated tandem devices in the past with physical TRJ.^36–39^ However, the ongoing work is carried out using the SCAPS-1D simulator, which does not support the TRJ layer and only supports a maximum of seven semiconducting layers. Therefore, the filtered spectrum followed by the current matching technique is utilized to design and investigate the PVK–Si monolithic tandem solar cell. We placed all the physical layers in the top and bottom subcell as suggested by.^24^ It is worth noting that understanding carrier dynamics inside the device without investigating the energy bands is a complicated task, which is not revealed in the experimental work.^24^ Therefore, this work addresses the problem, and corresponding data is reported in Fig. 10. The EBD for top and bottom subcells is obtained individually and stacked together to form the entire EBD of the tandem device, as depicted in Fig. 10. The interface, *i.e.*, zero value on the *x*-axis, shows the interface between the top and bottom subcell and reveals the tunnel recombination phenomena. An electron from ITO combines with the hole in Me-4PACz to maintain the current matching. Although such recombination is not considered in this work owing to the limitations of SCAPS-1D, the EBD data showed that the experimentally fabricated device reported in ref. 24 has a perfect TRJ layer with minimal recombination losses. The movement of charge carriers in the top and bottom subcell is also highlighted better to understand the carrier movement in the tandem device.

**Fig. 11:**
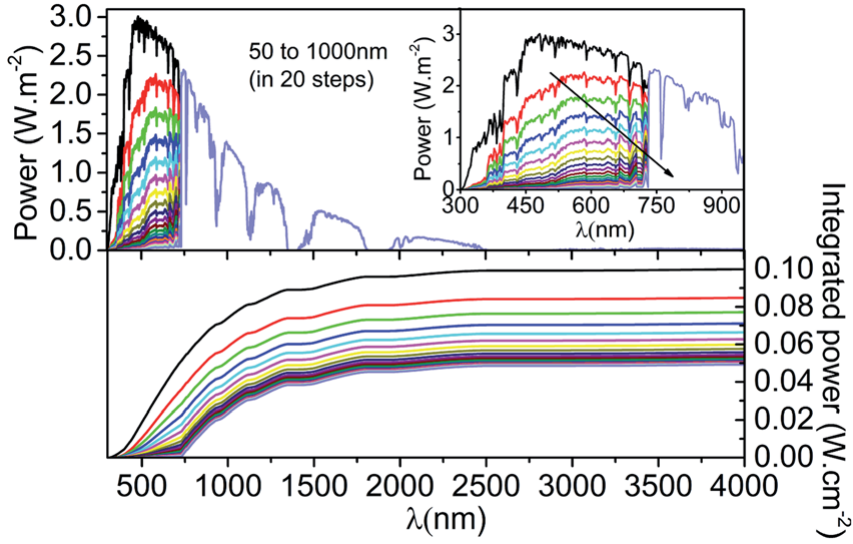
Filtered spectrum and integrated power density transmitted by top cell with different absorber layer thickness.

In series-connected tandem solar cells, equal current flows through both the devices and the cell with lower *J*_SC_ value in standalone configuration limits the tandem *J*_SC_, whereases net tandem *V*_OC_ is driven by the sum of the individual *V*_OC_ of top and bottom subcell. Enhancement in *V*_OC_ has the potential to lead the conversion efficiencies more than a single junction limit. The tandem device is designed using the current matching technique to connect the top and bottom cells. The standard global AM1.5 spectrum is illuminated on the wide-bandgap perovskite top cell to absorb the high-energy photons. While to conceive the physical presence of the top cell over the bottom cell, the bottom cell is supplied with the transmitted spectrum by the top cell to absorb the low-energy photons (for which the top cell was transparent). Additionally, Me-4PACz(p)/ITO-based ideal TRJ layer is also considered to account for parasitic absorption losses associated with TRJ while recombination losses are ignored. The filtered spectrum at different perovskite thicknesses is calculated by the following equation.^40,41^
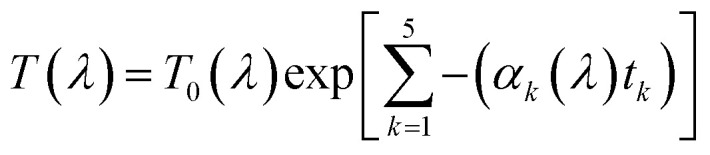
where *k* = 1, 2, 3, 4, and 5 are for IZO, SnO_2_, C_60_, perovskite, and Me-4PACz. Further, *α* is the absorption coefficient, and *t* is the thickness of the material. Twenty (20) different spectrum files are created and stored in the spectrum folder of SCAPS-1D, and all of them are used to conceive the presence of top cells with thickness absorber layer thickness for tandem simulation. The filtered spectrum and integrated power at different absorber layer thicknesses in the top cell are also reported in Fig. 11. The standard AM1.5 data is also reproduced in Fig. 11 to compare the AM1.5 spectrum and filtered spectrum. The filtered spectrum clearly shows that as the thickness of perovskite increases, the transmitted power to the bottom cell reduces considerably precisely for the wavelength below 738 nm. This reduction in transmitted power at thick perovskite is possessed by direct reliance on absorption and thickness (up to certain thickness). It is important to note that the absorber layer thickness as low as 50 nm significantly reduced the integrated power of the AM1.5 spectrum from 0.1 W cm^−2^ to 0.0492 W cm^−2^.

**Fig. 12:**
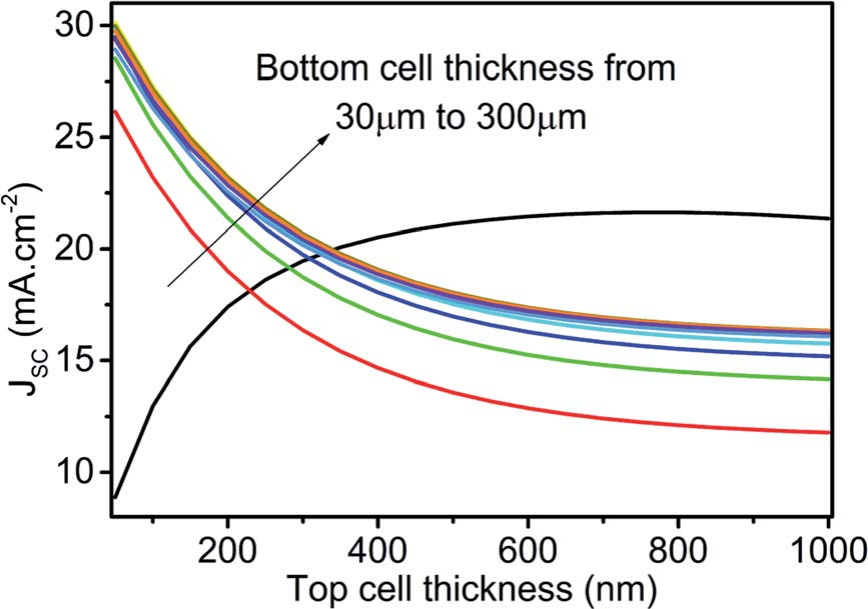
Current matching curve with varied thicknesses of absorber layer in top and bottom subcell. Top cell simulated under AM1.5 spectrum and bottom cell is stimulated by the top cell under filtered spectrum at respective absorber layer thickness.

**Fig. 13:**
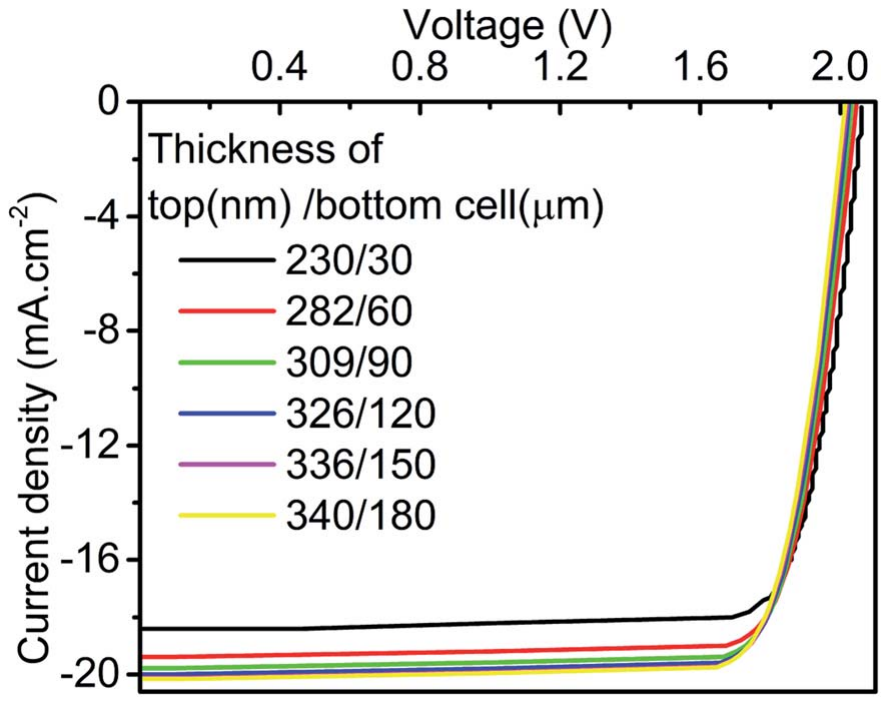
Constructed tandem *J*–*V* curve with thicknesses corresponding to first six current matching points.

As already stated, equal current in the top and bottom subcell is the essential criteria for monolithic tandem solar cells. Therefore, the thickness of the top and bottom subcell is varied simultaneously to obtain the thickness value for current matching. To find the current matching points, top cell thickness varies from 50 nm to 1000 nm, and bottom cell thickness ranges from 30 microns to 300 microns to obtain the *J*_SC_ as reported in Fig. 12. The top cell is simulated under the AM1.5 spectrum, and the bottom cell is simulated under the influence of the filtered spectrum. Therefore, the variation in *J*_SC_ for the bottom subcell is the consequence of absorber layer thickness in the top and bot subcell, whereases the change in top cell *J*_SC_ solely depends on absorber layer thickness in the top cell. Ten (10) intersecting, *i.e.*, equal current points, are observed; however, current-matched *J*_SC_ saturation is also observed for the current matching at higher thicknesses. Therefore, only the first six intersection points are selected to construct the tandem *J*–*V* curve and PV parameters. The voltages under the filtered spectrum of both cells are summed together at equal current points to get the tandem *J*–*V* curve, as shown in Fig. 13. The tandem *J*–*V* curves are constructed using the scripting features of SCAPS-1D and the code for the same is provided in ESI file.†

**Fig. 14:**
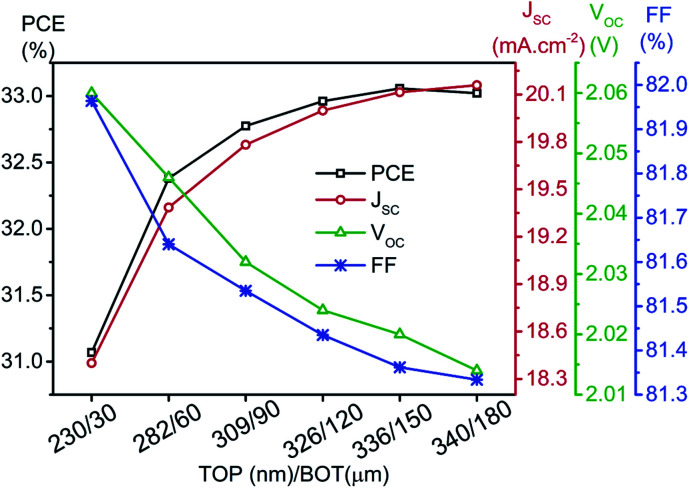
PVK–Si tandem solar cell parameters with thicknesses corresponding to the first six current matching points.

Fig. 13 shows the *J*–*V* curve of the tandem device, which is relatively better in comparison to the standalone condition in terms of *V*_OC_. The final PV parameters at a thickness corresponding to six current matching points, which are obtained in our work, are illustrated in Fig. 14. The improvement in the values of tandem *J*_SC_ can be observed in the tandem device at higher current matching points. Data also showed a marginal reduction in tandem *V*_OC_ and FF. Further, the tandem device is optimized with top/bottom subcell thickness of 336 nm/150 μm owing to the saturation and marginal reduction in PCE for top/bottom subcell thickness beyond this point (Fig. 14). The maximum value of *J*_SC_ is 20.15 mA cm^−2^ at 340 nm (top)/180 micron (bottom), which is quite similar to the limiting top cell *J*_SC_. The utilised spectrum files for the simulation of the optimized tandem device are shown in Fig. S1.†

**Fig. 15:**
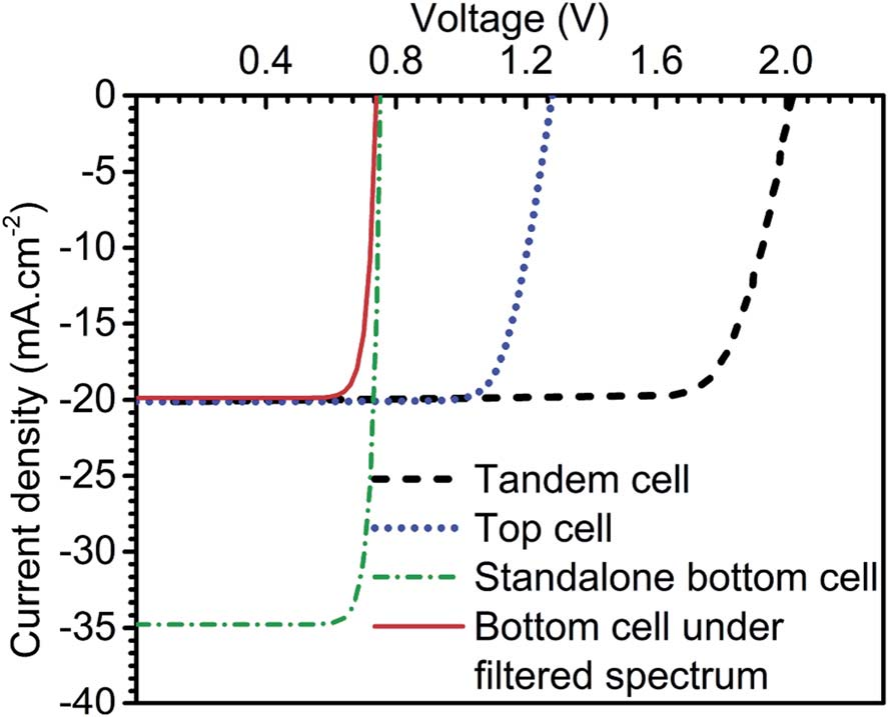
Illuminated *J–V* curve of standalone top and bottom subcell at optimised thickness, bottom cell under filtered spectrum by top cell and final tandem cell.

Furthermore, the tandem *V*_OC_ and FF values are 2.02 V and 81.9%, respectively. These PV parameters contributed to 33.1% tandem conversion efficiency in PVK–Si tandem solar cells. This section is concluded with illuminated *J*–*V* curve of the top compartment (336 nm), standalone bottom cell (150 μm), bottom cell (150 μm) under the filtered spectrum, and final PVK (336 nm)–Si (150 μm) monolithic tandem solar cell as demonstrated in Fig. 15. The summary of the PV parameters is outlined in Table 1.

**Table 1:** PV parameters summary of the devices considered in this subsection

Device	*V*_OC_ (V)	*J*_SC_ (mA cm^−2^)	FF (%)	PCE (%)
Top cell (336 nm)	1.28	20.11	79.90	20.58
Bottom cell (150 μm)	0.75	34.76	85.41	22.35
Bottom cell under filtered spectrum by top cell (336 nm)	0.72	19.83	85.08	12.15
PVK–Si monolithic tandem cell | top (336 nm)/bottom (150 μm)	2.02	20.11	81.36	33.05

## 4. Conclusions

This work presents an extensive device simulation of two-terminal monolithic perovskite–silicon (PVK–Si) tandem solar cells to achieve 33% conversion efficiency. Initially, the optimization of Me-4PACz hole transport layer-based enhanced hole extraction perovskite top cell and silicon-based bottom cell is carried out to deliver standalone conversion efficiency of 21.3% and 22.5%, respectively. After that, both the cells are integrated to form PVK–Si tandem solar cells. Filtered spectrum followed by current matching technique is utilized to simulate and optimize the tandem solar cell. The careful examination of the designed tandem solar cell is also carried out using the energy band diagram to understand the carrier dynamics. The PVK–Si tandem solar cell under consideration delivered 33% conversion efficiency with 336 nm and 150 μm thick absorber layer-based top and bottom subcell. The rest of the PV parameters are as follows *J*_SC_ (20.11 mA cm^−2^), *V*_OC_ (2.02 V), and FF (81.36%). The detailed study of the PVK–Si tandem design reported in the current study would open the path for the development of high-efficiency tandem solar cells in the future. The influence of the physical presence of tunnel recombination junction can be done in the future.

## Availability of data and material

The data that support the findings of this study shall be made available from the corresponding author upon reasonable request.

## Conflicts of interest

The authors declare no potential conflict of interest, financially or non-financially, directly or indirectly related to the work under consideration.

## Supplementary Material

RA-011-D1RA06250F-s001
