# Supplementary material for: Investigations aimed at producing 33% efficient perovskite–silicon tandem solar cells through device simulations
Source: RSC Adv. 2021 Nov 19;11(59):37366–74. doi: 10.1039/d1ra06250f (PMC9043820; doi:10.1039/d1ra06250f)
Supplement: RA-011-D1RA06250F-s001 [file RA-011-D1RA06250F-s001.pdf]

Electronic Supplementary Material (ESI) for RSC Advances.
This journal is © The Royal Society of Chemistry 2021

Supplementary Information

Investigations Aimed at Producing 33% Efficient Perovskite-Silicon Tandem Solar Cell Through Device Simulations

Nikhil Shrivastav[a], Jaya Madan[a], Rahul Pandey[a] and Ahmed Esmail Shalan[b,c]

[a] *VLSI Centre of Excellence, Chitkara University Institute of Engineering and Technology,* Chitkara University, Punjab, India
[b] *BCMaterials, Basque Center for Materials, Applications and Nanostructures, Martina Casiano, UPV/EHU Science Park, Barrio Sarriena s/n, Leioa 48940, Spain.*
[c] *Central Metallurgical Research and Development Institute (CMRDI), P. O. Box 87, Helwan, Cairo 11421, Egypt.*

Contents

1. Table S1
2. Table S2
3. Table S3
4. Spectrum files utilised for the tandem simulation
5. Script to calculate tandem J-V curve
6. References

1. Table S1

Table S1. The listed materials are used for the simulation of the device, and all the data related to its electrical and optical property is taken from already published papers [1-15].

**Properties**	Top Cell					Bottom Cell				
	**TL1**	**TL2**	**TL3**	**TL4**	**TL5**	**BL1**	**BL2**	**BL3**	**BL4**	**BL5**
**Bandgap (eV)**	3.5	3.6	1.7	1.68	3.3	3.5	1.4	1.74	1.12	1.74
**Electron affinity (eV)**	4.5	4.5	4.5	4.5	2.8	4.6	3.94	3.8	4.05	3.80
**Dielectric Constant**	10	9	6	10	10	8.9	11.9	11.9	11.9	11.9
**CB effective density (/cm³)**	1×10^{19}	2.2×10^{19}	1×10^{19}	1×10^{19}	1×10^{19}	2.2×10^{19}	2.8×10^{19}	1×10^{20}	2.8×10^{19}	1×10^{20}
**VB effective density (/cm³)**	1×10^{19}	1.8×10^{19}	1×10^{19}	1×10^{19}	1×10^{19}	1.8×10^{19}	1.4×10^{19}	1×10^{20}	1.04×10^{19}	1×10^{20}
**Electron mobility (cm²/Vs)**	1×10^{-2}	1×10^{2}	5×10^{1}	2×10^{1}	6×10^{0}	10	1.4×10^{3}	2×10^{1}	1.4×10^{3}	2×10^{1}
**Hole mobility (cm²/Vs)**	1×10^{-3}	2.5×10^{1}	5×10^{1}	2	2.4×10^{1}	10	4.5×10^{2}	5	4.5×10^{2}	5
**N_D (/cm³)**	1×10^{18}	1×10^{18}	1×10^{18}	1×10^{16}	0	1×10^{19}	1×10^{20}	1×10^{6}	5×10^{16}	1×10^{6}
**N_A (/cm³)**	0	0	0	0	1×10^{18}	0	0	1×10^{6}	0	1×10^{20}
TL1, TL2, TL3, TL4 and TL5 are IZO, SnO_2, C_{60}, perovskite and Me-4PACz, respectively.										
BL1, BL2, BL3, BL4 are BL5 are ITO, n-nc-SiO_x, i-a-Si:H, n-c-Si and p-a-Si:H, respectively.										

2. Table S2

Table S2. Details of interface defect parameters used during simulations.

Properties	Top cell interface defects				Bottom cell interface defects			
	**IZO / SnO2**	**SnO2 / C60**	**C60 / perovskite**	**perovskite/ Me-4PACz**	**i-a-Si:H/ n-nc-SiO_x**	**n-c-Si / i-a-Si:H**	**i-a-Si:H/ n-c-Si**	**p-a-Si:H /i-a-Si:H**
**Defect type**	neutral	neutral	neutral	neutral	neutral	neutral	neutral	neutral
**Capture cross section electrons(cm^2)**	1×10^{-19}	1×10^{-19}	1×10^{-19}	1×10^{-19}	1×10^{-15}	1×10^{-15}	1×10^{-19}	1×10^{-19}
**Capture cross section holes (cm^2)**	1×10^{-19}	1×10^{-19}	1×10^{-19}	1×10^{-19}	1×10^{-15}	1×10^{-15}	1×10^{-19}	1×10^{-19}
**Energetic distribution**	single	single	single	single	single	single	single	single
**Reference for defect energy level Et**	Above the highest E_v	Above the highest E_v	Above the highest E_v	Above the highest E_v	Above the highest E_v	Above the highest E_v	Above the highest E_v	Above the highest E_v
**Energy level w.r.t. reference (eV)**	.600	.600	.600	.600	.600	.600	.600	.600
**Total density (1/cm^2)**	1×10^1	1×10^1	1×10^1	1×10^1	1×10^1	1×10^1	1×10^1	1×10^1

3. Table S3

Table S3. Details of bulk defects parameters considered in intrinsic and p-type a-Si:H layers.

i-a-Si:H	p-a-Si:H
**Defect 1**<br>charge type: amphoteric: [+/0, 0/-]<br>total density (1/cm3): Uniform 8.000e+16<br>grading Nt(y): uniform<br>energy distribution: Gauss; Et = [0.70; 0.50] eV below E_C; Ekar = 0.29 eV	**Defect 1**<br>charge type: amphoteric: [+/0, 0/-]<br>total density (1/cm3): Uniform 8.000e+14<br>grading Nt(y): uniform<br>energy distribution: gauss; Et = [0.70; 0.50] eV below EC; Ekar = 0.29 eV
**Defect 2**<br>charge type: acceptor: [0/-]<br>total density (1/cm3): Uniform 1.000e+16<br>grading Nt(y): uniform<br>energy distribution: CB tail; Et = 0.01 eV below E_C; Ekar = 0.10 eV	**Defect 2**<br>charge type: acceptor: [0/-]<br>total density (1/cm3): Uniform 1.000e+15<br>grading Nt(y): uniform<br>energy distribution: CB tail; Et = 0.01 eV below EC; Ekar = 0.10 eV
**Defect 3**<br>charge type: donor: [+/0]<br>total density (1/cm3): Uniform 1.000e+16<br>grading Nt(y): uniform<br>energy distribution: VB tail; Et = 0.01 eV above EV; Ekar = 0.10 eV	**Defect 3**<br>charge type: donor: [+/0]<br>total density (1/cm3): Uniform 1.000e+16<br>grading Nt(y): uniform<br>energy distribution: VB tail; Et = 0.01 eV above EV; Ekar = 0.10 eV

4. Spectrum files utilised for the tandem simulation

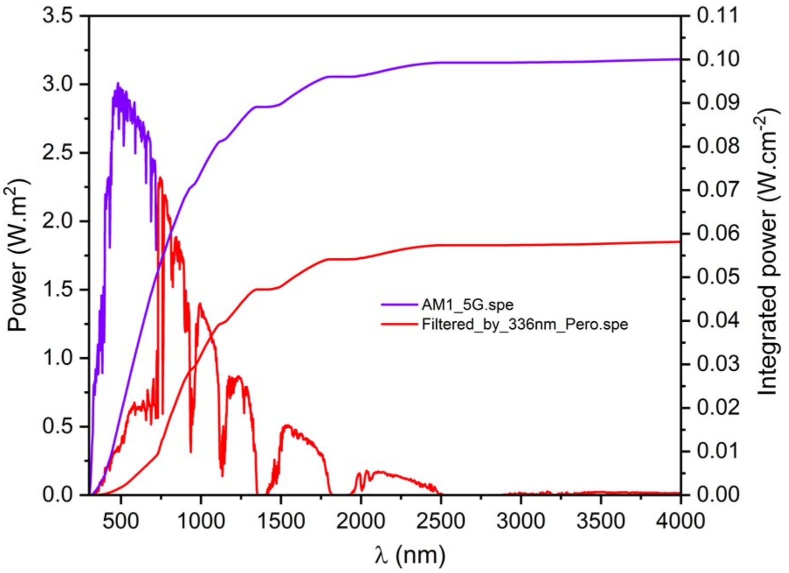

Fig. S1 Spectrum files used during tandem simulation.

5. Script to calculate tandem J-V curve.

```
// SCAPS script
clear all // take a clean start
load definitionfile 08062021_Nikhil 1new.def // or your def file of the top cell (need to be in the scaps/def folder)
action spectrumfile AM1_5G.spe // load the standard AM 1.5G spectrum
set layer4.thickness 0.336 // locate the layer number for the active absorber layer and set the thickness
calculate singleshot // top cell simulation under AM1.5 G spectrum
get iv xy // for top cell: V is stored in xvector, I in yvector
get characteristics.jsc yvalue // jsc is stored in yvalue
set scriptvariable.yvalue yvector[0] // the first calculated J value is in yvalue
math scalarabs yy  // the first calculated |J| value is in yvalue
// show scriptvariables

load definitionfile 08062021_HIT_for_Bottom_Nikhil.def // or your def file of the bottom cell (need to be in the scaps/def folder)
action spectrumfile Filtered_by_336nm_Pero.spe // load the filtered spectrum by the top cell
set layer3.thickness 150 // locate the layer number for the active absorber layer and set the thickness
calculate singleshot // bottom cell simulation under filtered spectrum
get iv zu // for bottom cell: V is stored in uvector, I in zvector
get characteristics.jsc uvalue // jsc is stored in uvalue

set scriptvariable.vvalue 0.002 // start varying Jtandem from Jsc(top) + this increment (in mA/cm2) (should be > 0, not <= 0)
math scalarsubtract vvy // starting value for Jtandem
set scriptvariable.wvalue 0 // stop value for Jtandem
math filllinear vvector vvalue wvalue 100 // fill vvector = Jtandem with (here) 100 values from start to stop
// set length of v and w vectors
set scriptvariable.maxiteration nv
set scriptvariable.nw nv
math fillconstant w 0 nw
// show scriptvariables

// start the loop: vary Jtandem
loop start
// find the voltage of the top cell at this Jtandem (by interpolation); set it in xvalue
set scriptvariable.yvalue vvector[loopcounter]
math interpolate xXyY
// find the voltage of the bottom cell at this Jtandem (by interpolation); set it in zvalue
```

```
set scriptvariable.uvalue vvector[loopcounter]
math interpolate zZuU
// do the series connection: add the voltages of top and bottom cell and place the result in xvalue
math scalaradd xxz
set scriptvariable.wvector[loopcounter] xvalue // and set this xvalue in wvector
loop stop

// set suitable names the vectors, and plot them
set scriptvariable.xname Vtop (V)
set scriptvariable.yname Jtop (mA/cm2)
set scriptvariable.zname Vbottom (V)
set scriptvariable.uname Jbottom (mA/cm2)
set scriptvariable.wname Vtandem (V)
set scriptvariable.vname Jtandem (mA/cm2)
show scriptvariables
plot draw wv // the tandem cell I-V
plot draw xy // the top cell I-V
plot draw zu // the bottom cell I-V

// extracting the efficiency parameters of the tandem, and place themin the scalars xvalue, yvalue,... wvalue
math characteristics.voc xwv
math characteristics.jsc ywv
math characteristics.ff zwv
math characteristics.eta uwv
math characteristics.vmpp vwv
math characteristics.jmpp wwv
show scriptvariables
```

6. References

1. A. Al-Ashouri, E. Köhnen, B. Li, A. Magomedov, H. Hempel, P. Caprioglio, J. A. Márquez, A. B. Morales Vilches, E. Kasparavicius, J. A. Smith, N. Phung, D. Menzel, M. Grischek, L. Kegelmann, D. Skroblin, C. Gollwitzer, T. Malinauskas, M. Jošt, G. Matič, B. Rech, R. Schlatmann, M. Topič, L. Korte, A. Abate, B. Stannowski, D. Neher, M. Stolterfoht, T. Unold, V. Getautis and S. Albrecht, *Science*, 2020, **370**, 1300.
2. B. Chen, Z. J. Yu, S. Manzoor, S. Wang, W. Weigand, Z. Yu, G. Yang, Z. Ni, X. Dai, Z. C. Holman and J. Huang, *Joule*, 2020, **4**, 850-864.
3. L. Xiong, Y. Guo, J. Wen, L. Hongri, P. Qin and G. Fang, *Advanced Functional Materials*, 2018, DOI: 10.1002/adfm.201802757.
4. Y. Yao, X. Xu, X. Zhang, H. Zhou, X. Gu and S. Q. Xiao, *Materials Science in Semiconductor Processing*, 2018, **77**, 16-23.
5. M. Taguchi, A. Yano, S. Tohoda, K. Matsuyama, Y. Nakamura, T. Nishiwaki, K. Fujita and E. Maruyama, *Photovoltaics, IEEE Journal of*, 2014, **4**, 96-99.
6. J. Dréon, Q. Jeangros, J. Cattin, J. Haschke, L. Antognini, C. Ballif and M. Boccard, *Nano Energy*, 2020, **70**, 104495.
7. S. Kirner, L. Mazzarella, L. Korte, B. Stannowski, B. Rech and R. Schlatmann, *IEEE Journal of Photovoltaics*, 2015, **5**, 1-5.
8. E. Hoke, D. Slotcavage, E. Dohner, A. Bowring, H. Karunadasa and M. McGehee, *Chem. Sci.*, 2014, **6**.
9. Y. I. Prylutskyy, S. S. Durov, L. A. Bulavin, I. I. Adamenko, K. O. Moroz, I. I. Geru, I. N. Dihor, P. Scharff, P. C. Eklund and L. Grigorian, *International Journal of Thermophysics*, 2001, **22**, 943-956.
10. D. Datta and S. Kumar, *Journal of Applied Physics*, 2009, **106**, 074517.
11. P. Jeon, S. J. Kang, H. Lee, J. Lee, K. Jeong, J. Lee and Y. Yi, *Journal of Applied Physics*, 2012, **111**, 073711.
12. T. Golubev, D. Liu, R. Lunt and P. Duxbury, *AIP Advances*, 2019, **9**, 035026.
13. L. Xiong, Y. Guo, J. Wen, H. Liu, G. Yang, P. Qin and G. Fang, *Advanced Functional Materials*, 2018, **28**, 1802757.
14. B. Babu, I. Neelakanta Reddy, K. Yoo, D. Kim and J. Shim, *Materials Letters*, 2018, **221**, 211-215.
15. Y. Chen, Q. Meng, L. Zhang, C. Han, H. Gao, Y. Zhang and H. Yan, *Journal of Energy Chemistry*, 2019, **35**, 144-167.
